# Trends in the incidence of thymoma, thymic carcinoma, and thymic neuroendocrine tumor in the United States

**DOI:** 10.1371/journal.pone.0227197

**Published:** 2019-12-31

**Authors:** Chun-Hsiang Hsu, John K. Chan, Chun-Hao Yin, Ching-Chih Lee, Chyi-Uei Chern, Cheng-I Liao

**Affiliations:** 1 Division of Chest Medicine, Department of Internal Medicine, Kaohsiung Veterans General Hospital, Kaohsiung, Taiwan; 2 Division of Gynecologic Oncology, California Pacific & Palo Alto Medical Foundation Sutter Health, San Francisco, CA, the United States of Amaerica; 3 Department of Medical Education and Research, Kaohsiung Veterans General Hospital, Kaohsiung, Taiwan; 4 Department of Otolaryngology, Head and Neck Surgery, Kaohsiung Veterans General Hospital, Kaohsiung, Taiwan; 5 Department of Obstetrics and Gynecology, Kaohsiung Veterans General Hospital, Kaohsiung, Taiwan; 6 National Yang-Ming University School of Medicine, Taipei, Taiwan; Medical University of Graz, AUSTRIA

## Abstract

This study aimed to identify the trends in the incidence of thymic cancer, i.e., thymoma, thymic carcinoma, and thymic neuroendocrine tumor, in the United States. Data from the United States Cancer Statistics (USCS) database (2001–2015) and those from the Surveillance, Epidemiology, and End Results (SEER) database (SEER 9 [1973–2015], SEER 13 [1992–2015], and SEER 18 [2000–2015]) were used in this study. All incidences were per 100,000 population at risk. The trends in incidence were described as annual percent change (APC) using the Joinpoint regression program. Data from the USCS (2001–2015) database showed an increase in thymic cancer diagnosis with an APC of 4.89% from 2001 to 2006, which is mainly attributed to the significant increase in the incidence of thymoma and thymic carcinoma particularly in women. The incidence of thymic cancer did not increase from 2006 to 2015, which may be attributed to the increase in the diagnosis of thymic carcinoma from 2004 to 2015, with a concomitant decrease in thymoma from 2008 to 2015. Before declining, the age-specific incidence of thymic cancer peaked at ages 70–74 years, with a peak incidence at 1.06 per 100,000 population, and decreased in older age groups. The incidence of thymic cancer was higher in men than in women. Asian/Pacific Islanders had the highest incidence of thymoma, followed by black and then white people. The incidence of thymic carcinoma increased from 2004 to 2015, with a concomitant decrease in thymoma from 2008 to 2015. Asian/Pacific Islanders had the highest incidence of thymoma than other races.

## Introduction

Thymic cancers originate from the thymus and are classified as thymoma, thymic carcinoma, thymic neuroendocrine tumor (NET), and other types according to the World Health Organization (WHO) classification of tumors of the thymus. Among these tumors, thymomas, thymic carcinomas, and thymic NETs are referred to as thymic epithelial tumors [1].

Thymoma is a rare type of thymic tumor, although it is the most common anterior mediastinal tumor, accounting for up to 50% of all anterior mediastinal masses [2]. Thymic carcinomas are extremely rare, aggressive tumors, with a poorer prognosis than thymomas [3]. Thymic NET is even rarer, accounting for only 2%-5% of all thymic cancers [4,5]. The 5-year overall survival rates are approximately 90% for thymoma [6], 55% for thymic carcinoma [7], and between 28% and 75% for thymic NET [8,9].

In the US, Engels et al. have analyzed data from the National Cancer Institute’s Surveillance, Epidemiology, and End Results (SEER) database and have shown that the age standardized rate of thymoma is 0.13–0.15 per 100,000 population at risk [2,10]. Gaur et al. have found that the incidence of thymic NET is 0.02 per 100,000 population at risk [5].

In the Netherlands, de Jong et al. have used data from the Netherlands National Pathological Archives (PALGA) and the Netherland Cancer Registry Database. They have found that the incidence of thymoma is 0.22–0.26 per 100,000 population at risk and that of thymic carcinoma was lower at 0.03–0.06 per 100,000 population at risk [11].

In Europe, Siesling et al. have found that the age standardized rate of thymic epithelial tumors is 0.17 per 100,000 population at risk according to the RARECARE project. The incidence was highest in patients aged 65 years and older. Moreover, it was lowest in Northern and Eastern Europe and the UK and Ireland and was highest in Central and Southern European countries [12].

The incidence of thymoma was high in black people and Asian/Pacific Islander population, which indicates that genetics may play a role [10,13].

In Taiwan, according to the Cancer Registry Annual Report 2016, the incidence of thymic cancer was higher in men than in women (1.61 vs. 1.33 per 100,000 population at risk) [14].

In 2010, Engels has used data from the SEER 9 (1973–2006), SEER 13 (1992–2006), SEER 18 (1998–2002), and SEER*Stat for analysis [2]. The SEER is regularly updated twice a year. SEER*Stat uses the weighted least square method to calculate APC. However, it cannot identify when the changes in trends will occur. This characteristic is one of the main differences between the SEER*Stat and Joinpoint trend analysis software. In this study, we used the updated data as Joinpoint estimates, as previously described and shown in other studies.

## Materials and methods

Data about population-based cancer incidence in the United States are collected by the National Cancer Institute’s (NCI’s) SEER program and the Centers for Disease Control and Prevention’s National Program of Cancer Registries (NPCR). These data show nearly 100% coverage of the US population in the most recent time period. The combined data are the official source of federal statistics on cancer incidence and are referred to as the United States Cancer Statistics (USCS) [15]. Data from the USCS 2001–2015 public use database were used in this study. Trends in long-term incidence (1973–2015) were based on the SEER 9 Regs Research Databases (1973–2015, Connecticut, Hawaii, Iowa, New Mexico, Utah, and the metropolitan areas of Atlanta, Detroit, San Francisco–Oakland, and Puget Sound), representing approximately 9% of the US population [16]. Data from the SEER 13 Regs Research Databases (1992–2015, SEER 9 plus Los Angeles, San Jose–Monterey, rural Georgia, and the Alaska Native Tumor Registry), representing 13% of the US population, were used [17]. Moreover, data from the SEER 18 Regs Research Databases (2000–2015, SEER 13 plus Greater California, Greater Georgia, Kentucky, Louisiana, and New Jersey), covering 28% of the US Population, were utilized [18] (S1 Table).

The International Classification of Diseases for Oncology, 3rd edition (ICD-O-3) is the standard reference for coding the histology of tumors diagnosed in 2001 and thereafter. We used the ICD-O-3 topography code C37.9 and ICD-O-3 behavior code 3 to identify cases of malignant tumor in the thymus in the USCS and SEER databases. The USCS and SEER had different ICD-O-3 histology codes. In this study, we excluded the following ICD-O-3 histology codes according to the SEER guideline: 9050–9055 (mesothelioma), 9060–9091 (germ cell tumor), 9140 (Kaposi sarcoma), and 9550–9992 (lymphomas, leukemias, myelomas, lymphoreticular, and immunoproliferative diseases). Then, we used the ICD-O-3 histology code to identify cases of thymoma (8581–8585), thymic carcinoma (8020, 8023, 8033, 8070, 8082, 8123, 8140, 8200, 8260, 8310, 8430, 8480, 8560, 8576, and 8586), thymic neuroendocrine tumor (8013, 8041, 8045, 8240, and 8249), and other types of tumor (with different codes) according to the WHO Classification of Tumors of Endocrine Organs 4th edition. The USCS and SEER have been using ICD-O-3 since 2001. They also converted the old ICD-O-2 to ICD-O-3 before 2001 (S2 Table). All patients in our study had invasive thymic epithelial tumors, as reported in the SEER database.

All incidences were age-adjusted to the 2000 US standard population (18-year age groups-Census P25-1130) and were expressed per 100,000 population at risk, as calculated using the SEER*Stat software (version 8.3.5) of the National Cancer Institute [19]. The APC in rates and the average annual percent change (AAPC) were quantified using the Joinpoint Regression Program (version 4.6.0.0) of the National Cancer Institute [20,21].

Because the data were extracted from a public, deidentified database, this study did not require approval from an institutional review board.

## Results

**Fig 1:**
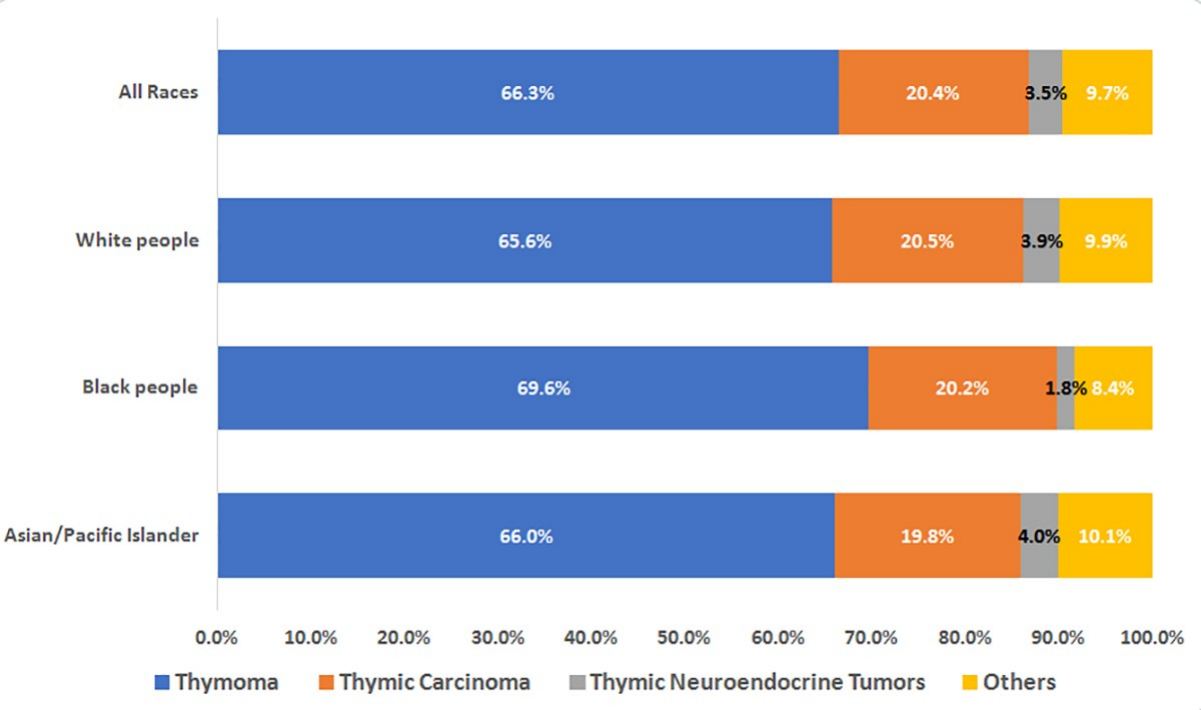
Proportion of each thymic cancer type in different races. SEER*Stat Database: NPCR and SEER Incidence—U.S. Cancer Statistics Public Use Database, Nov 2017 submission (2001–2015). Created on 9/15/2018; Surveillance, Epidemiology, and End Results (SEER) Program (www.seer.cancer.gov) SEER*Stat Database: Incidence—SEER 9 Regs Research Data, Nov 2017 Sub (1973–2015) <Katrina/Rita Population Adjustment>—Linked To County Attributes—Total U.S., 1969–2016 Counties, National Cancer Institute, DCCPS, Surveillance Research Program, released April 2018, based on the November 2017 submission.

**Fig 2:**
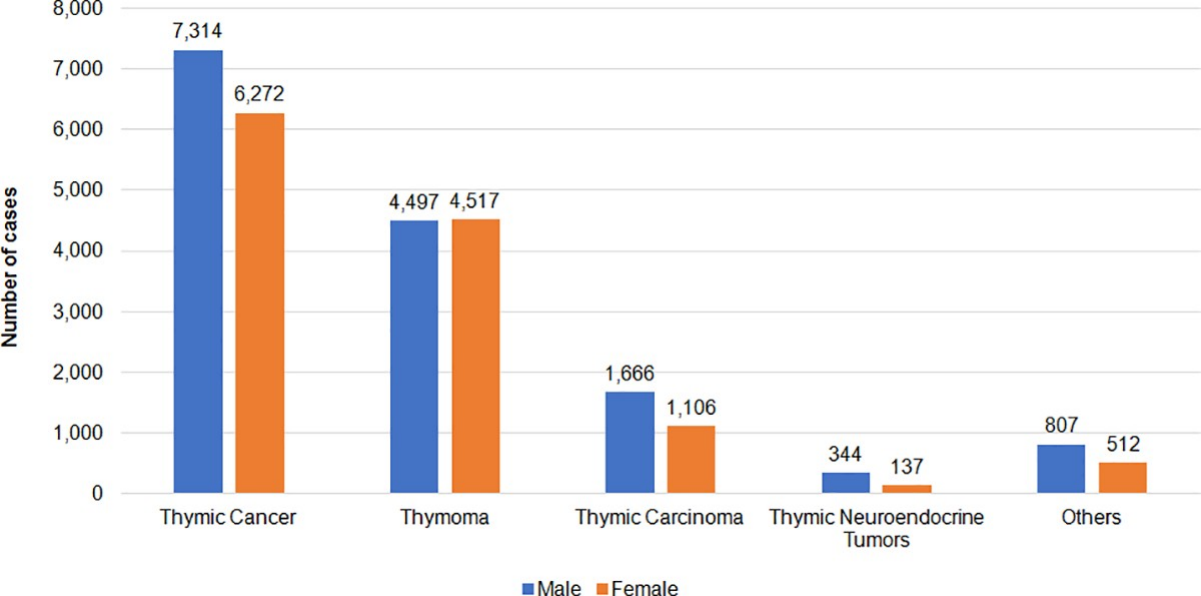
Number of patients of each thymic cancer type in different genders. SEER*Stat Database: NPCR and SEER Incidence—U.S. Cancer Statistics Public Use Database, Nov 2017 submission (2001–2015). Created on 9/15/2018; Surveillance, Epidemiology, and End Results (SEER) Program (www.seer.cancer.gov) SEER*Stat Database: Incidence—SEER 9 Regs Research Data, Nov 2017 Sub (1973–2015) <Katrina/Rita Population Adjustment>—Linked To County Attributes—Total U.S., 1969–2016 Counties, National Cancer Institute, DCCPS, Surveillance Research Program, released April 2018, based on the November 2017 submission.

Of the 13,586 patients diagnosed with thymic cancer, 9041 (66.3%), 2772 (20.4%), 481 (3.5%), and 1319 (9.7%) had thymoma, thymic carcinoma, thymic NET, and other types of tumors, respectively. The incidence of thymoma was higher in black people and the Asian/Pacific Islander population than in white people (Fig 1). The occurrence of thymic cancer was more common in men than in women. Meanwhile, the proportion of men and women with thymoma was similar (Fig 2).

### Trends in the incidence of thymic cancer

**Fig 3:**
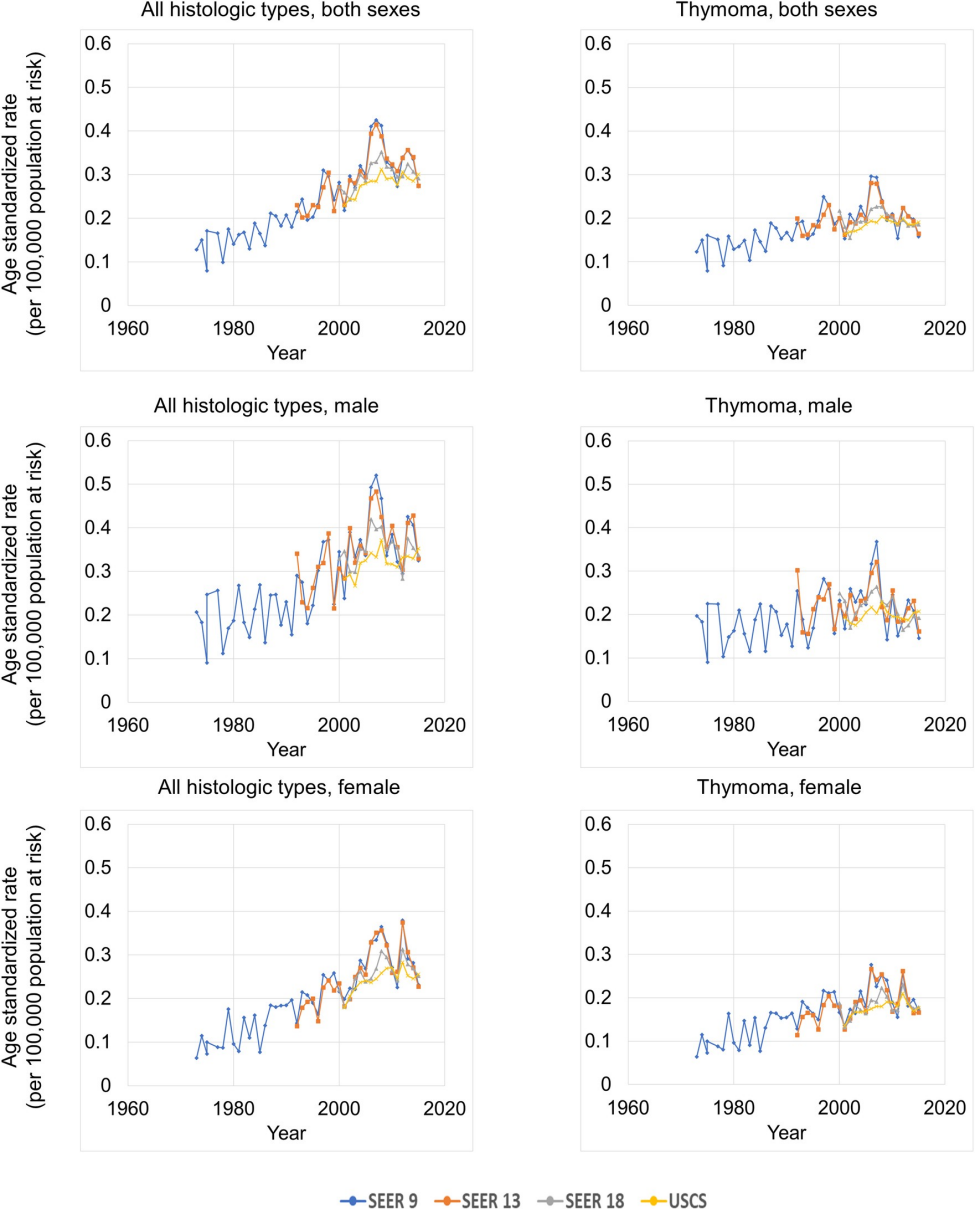
Thymic cancer age standardized rates over time, by data source, histological subtype and sex. SEER*Stat Database: NPCR and SEER Incidence—U.S. Cancer Statistics Public Use Database, Nov 2017 submission (2001–2015). Created on 9/15/2018; Surveillance, Epidemiology, and End Results (SEER) Program (www.seer.cancer.gov) SEER*Stat Database: Incidence—SEER 9 Regs Research Data, Nov 2017 Sub (1973–2015) <Katrina/Rita Population Adjustment>—Linked To County Attributes—Total U.S., 1969–2016 Counties, National Cancer Institute, DCCPS, Surveillance Research Program, released April 2018, based on the November 2017 submission.

Overall, the trends of age standardized rate in thymic cancer in the SEER 9, SEER 13, SEER 18, and USCS databases were increasing annually. Meanwhile, the incidences in the SEER 18 and USCS databases were similar, and those in the SEER 9 and SEER 13 databases were significantly higher than those in the SEER 18 and USCS databases, indicating possible regional differences (Fig 3; Table 1).

**Table 1:** Trends in thymic cancer age standardized rate by databases.

	The age standardized rate					Trend 1			Trend 2			Trend 3			
	1973	1992	2000	2001	2015	Years	APC	95% CI	Years	APC	95% CI	Years	APC	95% CI	AAPC
All histologic types, both sexes, all races															
SEER 9	0.13	0.21	0.28	0.22	0.27	1973–2008	3.3*	2.7–3.9	2008–2015	-2.9	-7.0–1.4				2.2*
SEER 13		0.23	0.27	0.23	0.27	1992–2007	4.1*	2.6–5.7	2007–2015	-2.1	-5.1–1.0				1.9*
SEER 18			0.27	0.26	0.29	2000–2008	3.6*	1.7–5.6	2008–2015	-1.8	-3.8–0.3				1.0
USCS				0.23	0.30	2001–2006	4.9*	1.8–8.1	2006–2015	0.1	-1.0–1.2				1.8*
All histologic types, male, all races															
SEER 9	0.21	0.29	0.35	0.24	0.33	1973–2015	2.2*	1.6–2.9							2.2*
SEER 13		0.34	0.31	0.28	0.33	1992–2015	1.6*	0.5–2.8							1.6*
SEER 18			0.33	0.35	0.34	2000–2015	0.3	-1.0–1.6							0.3
USCS				0.28	0.35	2001–2015	1.0*	0.1–2.0							1.0*
All histologic types, female, all races															
SEER 9	0.06	0.14	0.22	0.20	0.23	1973–2008	3.7*	2.9–4.4	2008–2015	-2.9	-7.8–2.3				2.6*
SEER 13		0.14	0.23	0.18	0.23	1992–2008	4.8*	2.9–6.8	2008–2015	-3.2	-8.3–2.1				2.3*
SEER 18			0.22	0.18	0.25	2000–2015	1.8*	0.4–3.2							1.8*
USCS				0.18	0.26	2001–2009	4.0*	1.9–6.2	2009–2015	-1.3	-3.9–1.5				1.7*
Thymoma, both sexes, all races															
SEER 9	0.12	0.19	0.20	0.15	0.15	1973–2007	2.1*	1.5–2.8	2007–2015	-4.2*	-8.1 - -0.1				0.9
SEER 13		0.20	0.20	0.16	0.16	1992–2007	2.4*	0.8–4.0	2007–2015	-3.6	-7.1–0.1				0.3
SEER 18			0.22	0.18	0.19	2000–2002	-11.8	-23.9–2.2	2002–2007	6.6*	1.9–11.4	2007–2015	-3.1*	-4.5 - -1.6	-1.2
USCS				0.16	0.19	2001–2008	2.9*	1.8–4.0	2008–2015	-0.9	-1.9–0.0				1.0*
Thymoma, male, all races															
SEER 9	0.20	0.25	0.23	0.17	0.15	1973–2015	0.8*	0.1–1.5							0.8*
SEER 13		0.30	0.22	0.20	0.16	1992–2015	-0.3	-1.6–1.0							-0.3
SEER 18			0.25	0.23	0.19	2000–2002	-15.7	-37.7–14.0	2002–2006	8.9	-7.2–27.8	2006–2015	-4.1*	-6.6 - -1.5	-2.5
USCS				0.19	0.21	2001–2015	0.4	-0.5–1.3							0.4
Thymoma, female, all races															
SEER 9	0.06	0.13	0.17	0.14	0.17	1973–2008	2.6*	1.8–3.4	2008–2015	-3.9	-9.4–2.0				1.5*
SEER 13		0.11	0.18	0.13	0.17	1992–2015	1.4*	0.2–2.7							1.4*
SEER 18			0.19	0.13	0.18	2000–2015	1.0	-0.5–2.6							1.0
USCS				0.14	0.18	2001–2012	2.5*	1.5–3.6	2012–2015	-5.4	-11.9–1.6				0.8

Abbreviation: CI, confidence interval; AAPC, average annual percent change over the most recent data years; APC, annual percent change

*The APC or AAPC is significantly different from zero (*P* < .05).

According to the USCS database, the incidence of age standardized rate of thymic cancer increased between 2001 and 2006, with an annual percentage change (APC) of 4.9% (p < 0.05). However, it did not change between 2006 and 2015, with an APC of 0.1% (p > 0.05).

### Histologic type

According to the USCS database, the age standardized rate of thymic cancer increased from 0.23/100,000 in 2001 to 0.30/100,000 in 2015, with AAPC of 1.8% (p < 0.05), and that of thymoma increased from 0.16/100,000 in 2001 to 0.19/100,000 in 2015, with an AAPC of 1.0% (p < 0.05). Meanwhile, the age standardized rate of thymic carcinoma also increased from 0.03/100,000 in 2001 to 0.07/100,000 in 2015, with an AAPC of 5.3% (p < 0.05), and that of thymic NET had a stable trend (from 0.01/100,000 in 2001 to 0.01/100,000 in 2015, AAPC = -2.3%, p > 0.05) (Table 2).

**Table 2:** Trends in thymic cancer age standardized rate by histologic types, sex, races in USCS, United States, 2001–2015.

	Incidence		Trend 1			Trend 2			2001–2015
	2001	2015	Years	APC	95% CI	Years	APC	95% CI	AAPC
All histologic Types, both sexes, all races	0.23	0.30	2001–2006	4.9*	1.8–8.1	2006–2015	0.1	-1.0–1.2	1.8*
Thymoma	0.16	0.19	2001–2008	2.9*	1.8–4.0	2008–2015	-0.9	-1.9–0.0	1.0*
Thymic carcinoma	0.03	0.07	2001–2004	15.7	-2.1–36.7	2004–2015	2.6*	1.0–4.3	5.3*
Thymic neuroendocrine tumor	0.01	0.01	2001–2015	-2.3	-4.7–0.2				-2.3
Sex									
Male	0.28	0.35	2001–2015	1.0*	0.1–2.0				1.0*
Female	0.18	0.26	2001–2009	4.0*	1.9–6.2	2009–2015	-1.3	-3.9–1.5	1.7*
Race									
White	0.21	0.25	2001–2005	5.8*	0.8–11.1	2005–2015	-0.3	-1.4–0.8	1.4
Black	0.33	0.50	2001–2015	2.0*	0.9–3.1				2.0*
Asian/Pacific Islander	0.37	0.60	2001–2015	2.4*	0.2–4.6				2.4*
Male									
Thymoma	0.19	0.21	2001–2015	0.4	-0.5–1.3				0.4
Thymic carcinoma	0.04	0.10	2001–2015	4.1*	2.5–5.8				4.1*
Thymic neuroendocrine tumor	0.02	0.01	2001–2015	-2.9*	-5.1 - -0.6				-2.9*
Female									
Thymoma	0.14	0.18	2001–2012	2.5*	1.5–3.6	2012–2015	-5.4	-11.9–1.6	0.8
Thymic carcinoma	0.02	0.05	2001–2009	7.9*	3.0–13.0	2009–2015	-1.7	-6.9–3.7	3.7*
Thymic neuroendocrine tumor	-	-	-	-	-				-

Abbreviation: CI, confidence interval; AAPC, average annual percent change over the most recent data years; APC, annual percent change

*The APC or AAPC is significantly different from zero (*P* < .05).

### Gender

According to the USCS database, the age standardized rate of thymic cancer in men increased from 0.28/100,000 in 2001 to 0.35/100,000, with an AAPC of 1.0% (p < 0.05), and that of thymic cancer in women also increased from 0.18/100,000 in 2001 to 0.26/100,000, with an AAPC of 1.7% (p < 0.05).

In men, the age standardized rate of thymoma did not significantly change from 0.19/100,000 in 2001 to 0.21/100,000 in 2015, with an AAPC of 0.4% (p > 0.05), and that of thymic carcinoma increased from 0.04/100,000 in 2001 to 0.10/100,000 in 2015, with an AAPC of 4.1% (p < 0.05). Meanwhile, the age standardized rate of thymic NET decreased from 0.02/100,000 in 2001 to 0.01/100,000 in 2015, with an AAPC of -2.9% (p < 0.05) (Table 2).

In women, the age standardized rate of thymoma remained stable from 0.14/100,000 in 2001 to 0.18/100,000 in 2015, with an AAPC of 0.8% (p > 0.05), and that of thymic carcinoma increased from 0.02/100,000 in 2001 to 0.05/100,000 in 2015, with an AAPC of 3.7% (p < 0.05). However, the age standardized rate of thymic NET could not be calculated due to the small number of cases (Table 2).

### Race

The Asian/Pacific Islanders have the highest incidence and increase in the incidence of thymic cancer (from 0.37/100,000 in 2001 to 0.60/100,000 in 2015, AAPC = 2.4%, p < 0.05), followed by black people (from 0.33/100,000 in 2001 to 0.50/100,000 in 2015, AAPC = 2.0%, p < 0.05) and white people (from 0.21/100,000 in 2001 to 0.25/100,000 in 2015, AAPC = 1.4%, p > 0.05) (Table 2).

### Age-specific incidence

**Fig 4:**
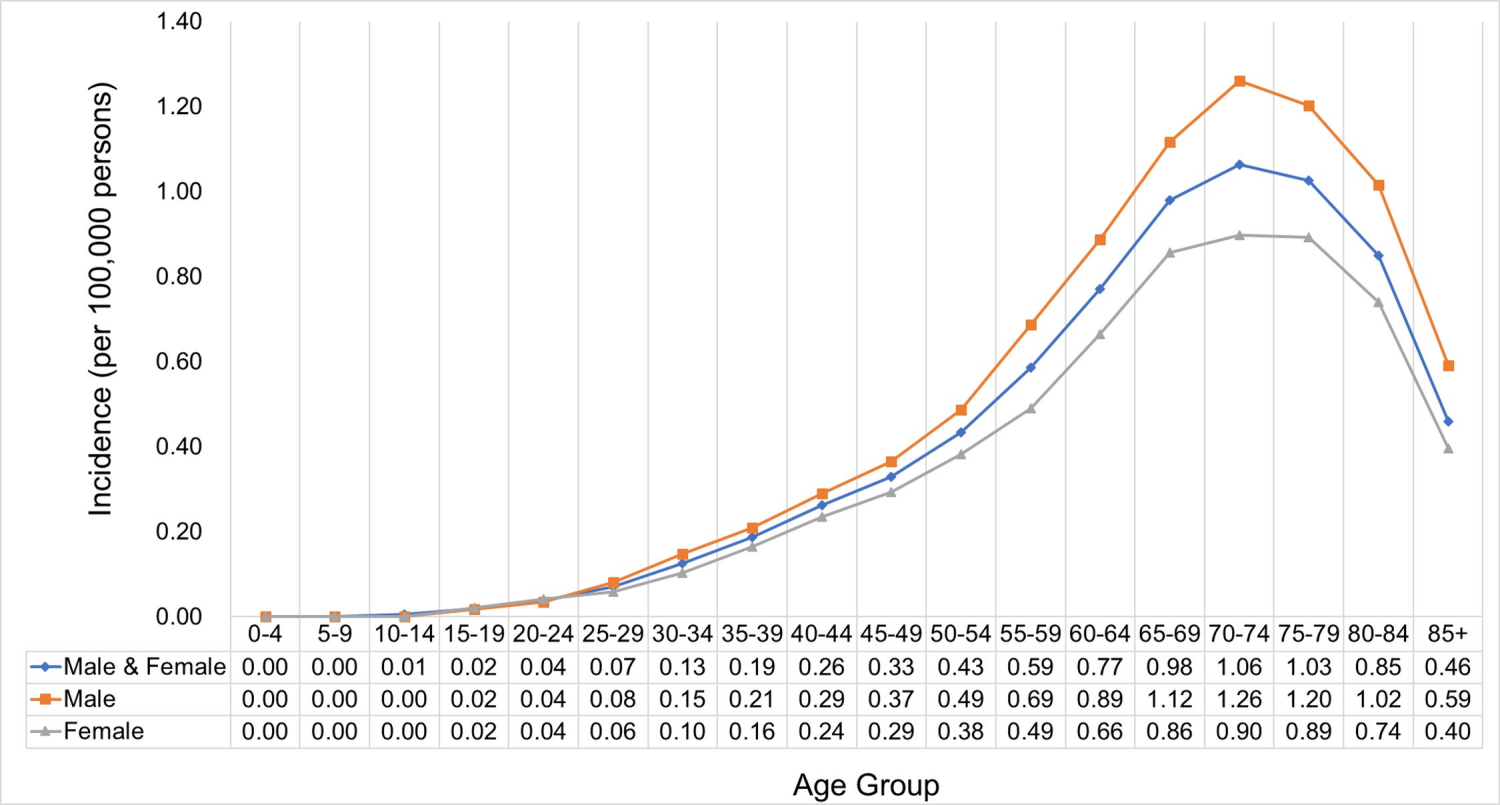
Age-specific incidence of thymic cancer by sex. SEER*Stat Database: NPCR and SEER Incidence—U.S. Cancer Statistics Public Use Database, Nov 2017 submission (2001–2015). Created on 9/15/2018; Surveillance, Epidemiology, and End Results (SEER) Program (www.seer.cancer.gov) SEER*Stat Database: Incidence—SEER 9 Regs Research Data, Nov 2017 Sub (1973–2015) <Katrina/Rita Population Adjustment>—Linked To County Attributes—Total U.S., 1969–2016 Counties, National Cancer Institute, DCCPS, Surveillance Research Program, released April 2018, based on the November 2017 submission.

The age-specific incidence of thymic cancer continually increased in the 70-74-year-old age group, with a peak incidence at 1.06/100,000, and decreased in the older age groups. The age standardized rate was higher in men than in women (Fig 4).

**Fig 5:**
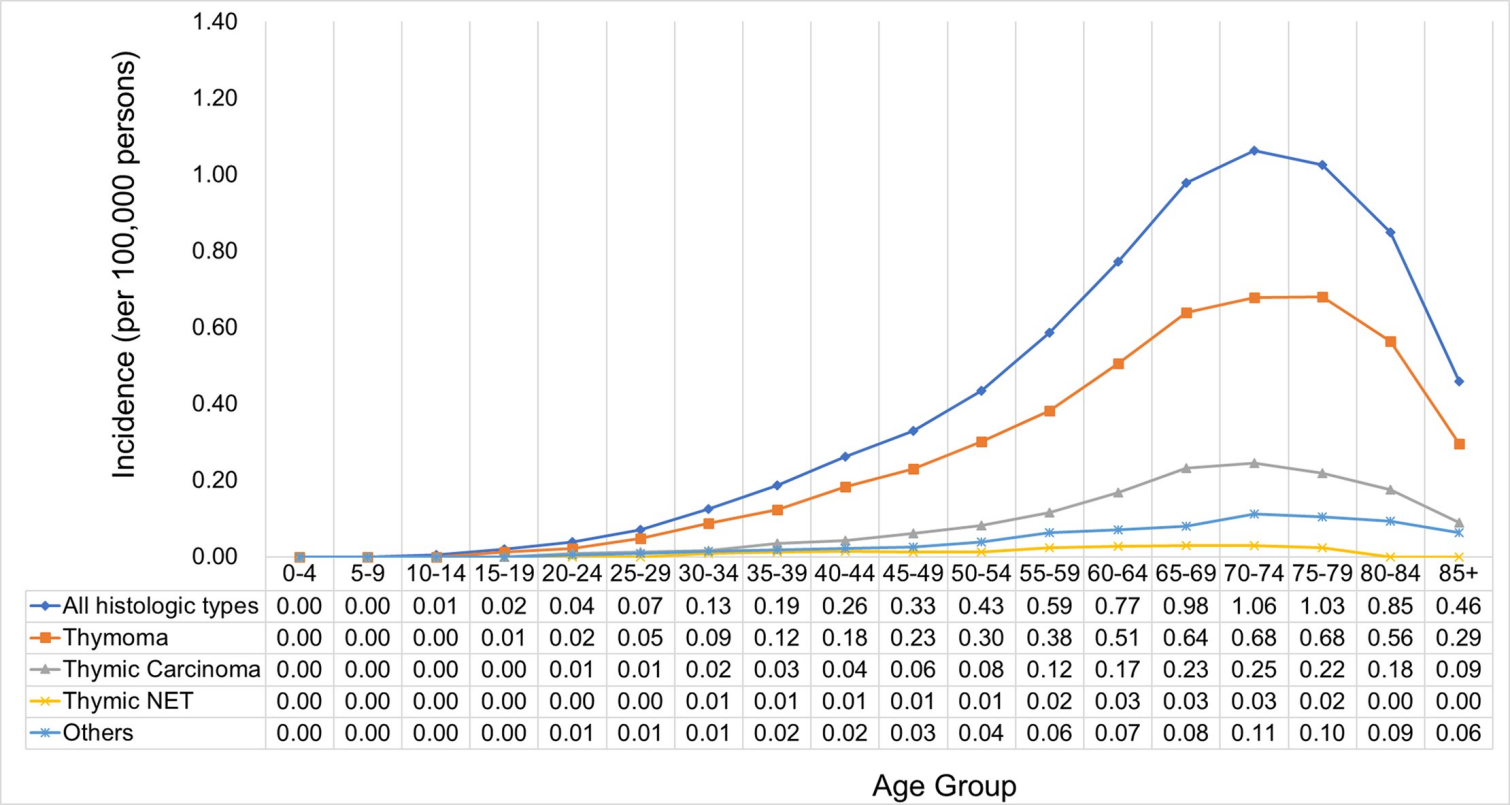
Age-specific incidence of thymic cancer by histologic type. SEER*Stat Database: NPCR and SEER Incidence—U.S. Cancer Statistics Public Use Database, Nov 2017 submission (2001–2015). Created on 9/15/2018; Surveillance, Epidemiology, and End Results (SEER) Program (www.seer.cancer.gov) SEER*Stat Database: Incidence—SEER 9 Regs Research Data, Nov 2017 Sub (1973–2015) <Katrina/Rita Population Adjustment>—Linked To County Attributes—Total U.S., 1969–2016 Counties, National Cancer Institute, DCCPS, Surveillance Research Program, released April 2018, based on the November 2017 submission.

Regarding the histologic type, thymoma has the highest incidence (peak at 0.68 per 100,000 population in the 70-79-year-old age group), followed by thymic carcinoma (peak at 0.25 per 100,000 population in the 70-74-year-old age group) and then thymic NET (peak at 0.03 per 100,000 population in the 60-74-year-old age group). Unlike in thymoma and thymic carcinoma, the peak age-specific incidence of thymic NET was observed in a younger age group (Fig 5).

**Fig 6:**
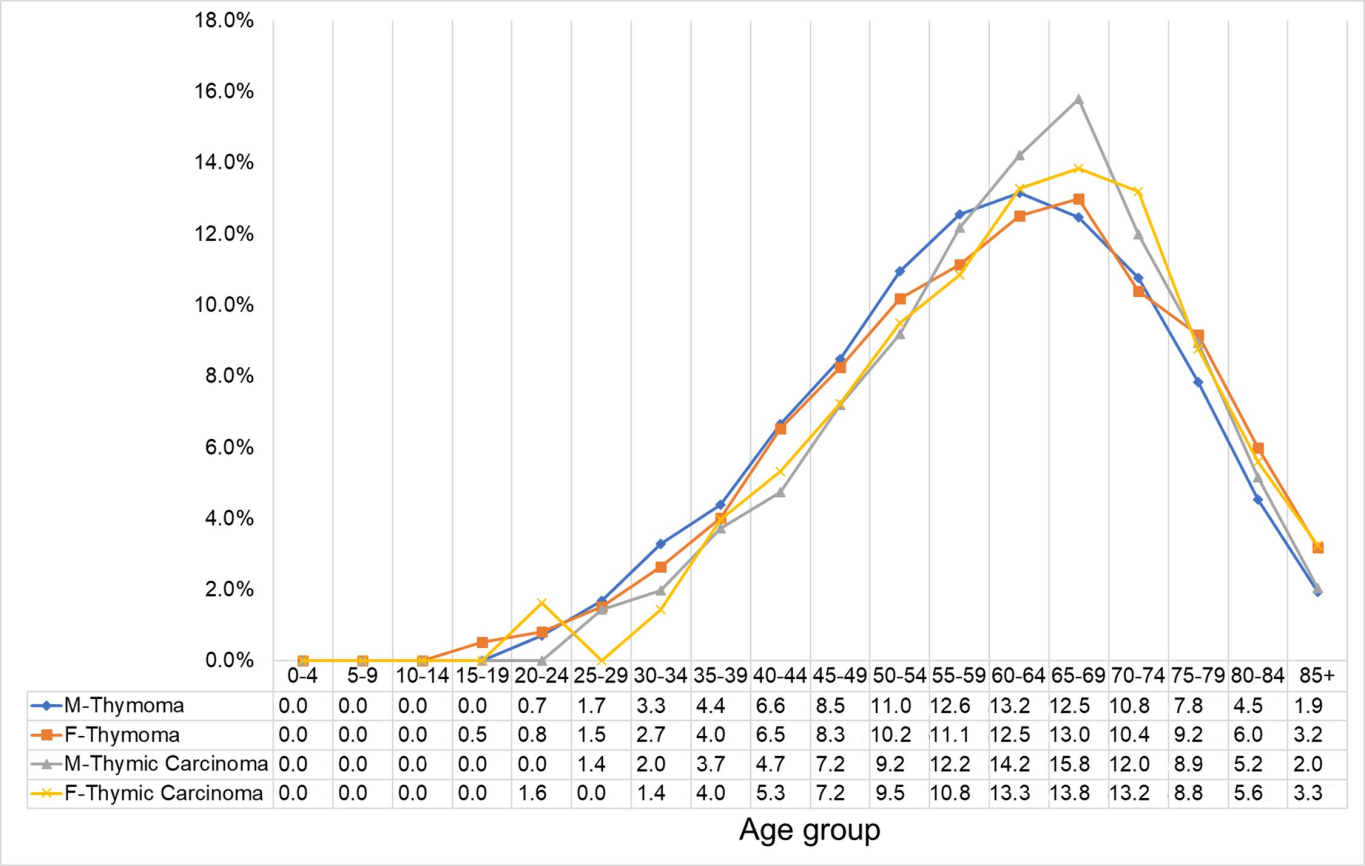
Case proportion of thymic cancer stratified by sex and histologic type. SEER*Stat Database: NPCR and SEER Incidence—U.S. Cancer Statistics Public Use Database, Nov 2017 submission (2001–2015). Created on 9/15/2018; Surveillance, Epidemiology, and End Results (SEER) Program (www.seer.cancer.gov) SEER*Stat Database: Incidence—SEER 9 Regs Research Data, Nov 2017 Sub (1973–2015) <Katrina/Rita Population Adjustment>—Linked To County Attributes—Total U.S., 1969–2016 Counties, National Cancer Institute, DCCPS, Surveillance Research Program, released April 2018, based on the November 2017 submission.

The highest proportion of cases were follows: 13.2%, 60-64-year-old men with thymoma; 15.8%, 65-69-year-old men with thymic carcinoma; 13.0%, 65-69-year-old women with thymoma; and 13.8%, 65-69-year-old women with thymic carcinoma. The sample size of the patients with thymic NET was extremely small to be included in the analysis (Fig 6).

## Discussion

In our study, the incidence of thymic carcinoma increased from 2004 to 2015, with a concomitant decrease in thymoma from 2008 to 2015. However, previous reports have not shown a change in incidence over time.

We hypothesized that this trend may be attributed to advancements in immunohistochemistry (IHC) technique and modification of the classification of thymic cancer.

### Advanced IHC technique

The diagnosis of thymic cancer is considered a challenge due to its heterogeneity and rarity. In recent years, the IHC technique has been significantly developed, and pathological protocols have been established [4,5,22, 23,24].

The routine assessment of IHC markers is recommended for thymomas and thymic carcinomas with ambiguous histology [1]. In recent years, several novel IHC markers expressed in most patients with thymic carcinomas have been discovered. Weissferdt A et al. have designed panels of several markers that can be used to differentiate thymomas from thymic carcinomas [25].

In the past, pathologists might not have enough experience in managing thymic cancer due to its rarity. Detterbeck et al. have found that some types of thymoma (WHO type B3) and thymic carcinoma are not well differentiated [23]. Primary thymic carcinoma is difficult to distinguish from metastatic carcinoma of other origin [23,25]. Thus, we hypothesized that advancements in IHC technique can help differentiate thymomas from thymic carcinomas, thereby leading to an increase in the number of patients diagnosed with thymic carcinomas.

### Modification of the classification of thymic epithelial tumor

Several different systems have been used in the classification of thymic epithelial tumor. In 1999, the WHO translated the terminologies of earlier systems into alternate classification systems based on the letters and numbers of thymoma, including types A, AB, B1, B2, B3, and C [26]. In 2004, the new WHO classification excluded type C thymoma and replaced it with thymic carcinoma [4].

A similar study that utilized the SEER program data has shown a decrease in the incidence of thymoma from 1997 to 2006, which can potentially reflect changes in the classification of thymic cancer [2].

The increase in the average age of the population might also had an impact on the decreased incidence of thymoma from 2008 to 2015. Thymoma in older patients may be underdiagnosed due to nonspecific presentation (such as weakness from myasthenia gravis), which may be neglected by clinicians [10]. If thymic capsule invasion of thymoma is not observed, physicians or pathologists often consider the condition as benign. Such cases are often missed in cancer registries, and the incidence of thymoma is low based on registry data [2].

### Age, gender, and race

Our study showed that the age-specific peak incidence of thymoma was observed in the 70-79-year-old age group, with a peak incidence at 0.68/100,000. This result is consistent with that of a prior study conducted by Engels et al. in the US from 1973 to 1998 [10]. The peak incidence in our study was in a younger age group in thymic NET at 60–74 years compared to that in thymoma at 70–79 years and thymic carcinoma at 70–74 years. A previous study has shown that the median age at diagnosis for thymic NETs is 59 years [27].

The age standardized rate of thymic cancer was higher in men than in women, which is consistent with a prior study [10].

The incidence of thymoma varied in terms of race. In this study, the incidence of thymoma was higher in black people than in white people in both genders. A recent literature has shown that the incidence is higher in black people than in white people (0.20/100,000 vs. 0.12/100,000), which indicates that genetics plays a role [2,10,13]. The incidence increased in white women diagnosed with thymoma from 2001 to 2012, with an APC of 2.5%.

### Strengths and limitations of the study

The present study had several limitations. Since this is a retrospective review, information bias exists, with possibility of missing or ambiguous data. A central pathology review was conducted, which might had resulted in significant variations in diagnosis among pathologists from different institutions.

To the best of our knowledge, this is the largest research that analyzed the trends in the incidence of thymic cancer. The USCS database is an extensive cancer registry with standardized data entry by highly trained cancer registrars. These data represent 97.0% of the entire US population, which is superior to other smaller, single-institution studies [28].

## Conclusion

The incidence of thymic carcinoma increased from 2004 to 2015, with a concomitant decrease in thymoma from 2008 to 2015. The trend may be primarily attributed to advancements in IHC technique and modification of the classification of thymic cancer.

Thymic NETs were initially classified as thymic carcinoma, and they have been incorporated into a new category of thymus tumors since 2015. Thus, long-term outcome and incidence rate should be followed-up.

The implications of this study are important for clinicians because thymic carcinoma is a progressive disease and less resectable and has poorer outcome than thymoma. The early identification of thymic carcinoma allows for prompt treatment.

The age-specific incidence was highest in 60-64-year-old men and 65-69-year-old women. This result should be taken into consideration when assessing a mediastinal mass in patients from these populations.

More studies must be conducted to assess the incidence, trends, and genomic analysis findings of thymic cancer in areas other than the US, such as Europe, Asia-Pacific regions, and Africa. Genetic factor may play a role in the pathogenesis of thymoma, causing racial differences in its incidence.

## Supporting information

S1 Table Data source. (DOCX)

S2 Table Histology code. (DOCX)
