# Supplementary material for: Trends in the incidence of thymoma, thymic carcinoma, and thymic neuroendocrine tumor in the United States
Source: PLoS One. 2019 Dec 31;14(12):e0227197. doi: 10.1371/journal.pone.0227197 (PMC6938371; doi:10.1371/journal.pone.0227197)
Supplement: S1 Table — (DOCX) [file pone.0227197.s001.docx]

**S1 table. Data source**

| Database | Year | Area |
| --- | --- | --- |
| SEER 9 Regs Research Data | 1973-2015 | Altanta, Connecticut, Detroit, Hawaii, Lowa, New Mexico, San Francisco-Oakland, Seattle-Puget Sound, Utah |
| SEER 13 Regs Research Data | 1992-2015 | SEER 9 Regs + Los Angeles, San Jose-Monterey, Rural Georgia and the Alaska Native Tumor Registry |
| SEER 18 Regs Research Data | 2000-2015 | SEER 13 Regs + Greater California, Greater Georgia, Kentucky, Louisiana, and New Jersey |
| USCS | 2001-2015 | 50 states |

Abbreviation: SEER,Surveillance, Epidemiology, and End Results; USCS,United States Cancer Statistics
