# Supplementary material for: Trends in the incidence of thymoma, thymic carcinoma, and thymic neuroendocrine tumor in the United States
Source: PLoS One. 2019 Dec 31;14(12):e0227197. doi: 10.1371/journal.pone.0227197 (PMC6938371; doi:10.1371/journal.pone.0227197)
Supplement: S2 Table — (DOCX) [file pone.0227197.s002.docx]

**S2 table. Histology Code**

| Cell Type | ICD-O-3 Topographical Code | Behavior Code | ICD-O-3  Morphological Code |
| --- | --- | --- | --- |
| Thymic Cancer | C37.9 | 3 | All, but exclude 9050-9055, 9060-9091, 9140, 9590-9992 |
| Thymoma | C37.9 | 3 | 8581-8585 |
| Thymic carcinoma | C37.9 | 3 | 8020, 8023, 8033, 8070, 8082, 8123, 8140, 8200, 8260, 8310, 8430, 8480, 8560, 8576, 8586 |
| Thymic neuroendocrine tumor | C37.9 | 3 | 8013, 8041, 8045, 8240, 8249 |

Abbreviation: ICD-O-3, International Classification of Disease for Oncology, version 3
